# A scoping review of emotions and related constructs in simulation-based education research articles

**DOI:** 10.1186/s41077-023-00258-z

**Published:** 2023-09-16

**Authors:** Byunghoon (Tony) Ahn, Meagane Maurice-Ventouris, Elif Bilgic, Alison Yang, Clarissa Hin-Hei Lau, Hannah Peters, Kexin Li, Deuscies Chang-Ou, Jason M. Harley

**Affiliations:** 1 Department of Surgery, McGill University, Montreal, Canada; 2 Department of Pediatrics, McMaster University, Hamilton, Canada; 3 McMaster Education Research Innovation and Theory (MERIT) program, Hamilton, Canada; 4 Research Institute of the McGill University Health Centre, Montreal, Canada; 5 Institute for Health Sciences Education, McGill University, Montreal, Canada; 6 Steinberg Centre for Simulation and Interactive Learning, McGill University, Montreal, Canada

**Keywords:** Emotion, Stress, Emotional intelligence, Simulation-based education, Medical education, Surgical education, Scoping review

## Abstract

**Background:**

While acknowledgement of emotions’ importance in simulation-based education is emerging, there are concerns regarding how education researchers understand the concept of emotions for them to deliberately incorporate emotionally charged scenarios into simulation-based education. This concern is highlighted especially in the context of medical education often lacking strong theoretical integration. To map out how current simulation-based education literature conceptualises emotion, we conducted a scoping review on how emotions and closely related constructs (e.g. stress, and emotional intelligence) are conceptualised in simulation-based education articles that feature medical students, residents, and fellows.

**Methods:**

The scoping review was based on articles published in the last decade identified through database searches (EMBASE and Medline) and hand-searched articles. Data extraction included the constructs featured in the articles, their definitions, instruments used, and the types of emotions captured. Only empirical articles were included (e.g. no review or opinion articles). Data were charted via descriptive analyses.

**Results:**

A total of 141 articles were reviewed. Stress was featured in 88 of the articles, while emotions and emotional intelligence were highlighted in 45 and 34 articles respectively. Conceptualisations of emotions lacked integration of theory. Measurements of emotions mostly relied on self-reports while stress was often measured via physiological and self-report measurements. Negative emotions such as anxiety were sometimes seen as interchangeable with the term stress. No inferences were made about specific emotions of participants from their emotional intelligence.

**Conclusions:**

Our scoping review illustrates that learners in simulation-based education are most often anxious and fearful. However, this is partially due to medical education prioritising measuring negative emotions. Further theoretical integration when examining emotions and stress may help broaden the scope towards other kinds of emotions and better conceptualisations of their impact. We call for simulation education researchers to reflect on how they understand emotions, and whether their understanding may neglect any specific aspect of affective experiences their simulation participants may have.

**Supplementary Information:**

The online version contains supplementary material available at 10.1186/s41077-023-00258-z.

## Introduction

Training for delivering better patient outcomes requires understanding emotions—they are pervasive throughout healthcare environments for both physicians and trainees [1]. Moreover, recent medical education research has highlighted the roles and potential impact of emotions in training future doctors [2, 3]. Not only can emotionally charged scenarios sway clinical decisions [4], but being able to understand, monitor, and manage one’s own and others’ emotions (i.e. emotional intelligence) is deemed crucial for developing core competencies physicians require [5] as it relates to desirable aptitudes such as resiliency against burnout [6], leadership [7], and communication [8, 9]. In sum, medical education can help prepare learners to be emotionally resilient and better emotionally attuned to one another and their patients. Therefore, advancing these goals and informing practice requires a better understanding of medical trainees’ emotions during medical education, including simulations.

Simulation-based education (SBE) is no exception to emotions’ pervasiveness: a recent conceptual review by LeBlanc and Posner highlighted how SBE can be “rife with emotional situations” [10], (p.6). Indeed, the ubiquitous nature of emotions in medicine, including in SBE has been echoed previously [1, 11] and its potential impact on healthcare practice and education has been empirically documented in various domains, including diagnostic reasoning [12], patient-physician communication [13], and patient safety issues related to medical errors [14, 15].

LeBlanc and Posner’s review [10] also underscored how simulation educators’ various beliefs regarding the role of emotions are often based on personal experiences—one concern regarding this is whether educators can consistently be “thoughtful and deliberate” [10], (p.5) when incorporating emotional experiences into SBE scenarios. This can be especially so when simulation educators conceptualise and use the term emotions in everyday and professional discourse. For example, McNaughton’s discourse analysis shows how educators can view emotions not only as a “series of biological and neurochemical responses”, but also as “skills that can be learned” (i.e. emotional intelligence; EI), and “a set of practices that are constructed by social, cultural, and political arrangements” [11], (p.73). To put the concern another way: are medical educators understanding emotions as something interchangeable to EI? Will they distinguish something like stress (also a product of “series of biological and neurochemical responses”) from emotions? To truly be purposeful in incorporating emotions in SBE, educators should be aware of how they conceptualise emotions, and why they subscribe to their understanding.

Integrating a theoretical framework into research is a straightforward way of advancing understanding of emotions (e.g. see Coppin & Sander [16]). While medical education research often lacks strong theoretical integration [17, 18], there have been calls for integrating frameworks such as the Control Value Theory of Achievement Emotions (CVT) [1, 2, 19], a prominent theoretical framework in educational psychology [20, 21]. CVT’s definition of emotion aligns with the consensus mainstream emotion theories and its definition can help educators distinguish emotions from other affective phenomena including moods and stress [19, 22]. CVT defines emotions as multi-componential psychological responses produced by coordinated affective, cognitive, motivational, and expressive processes. Emotions are described based on a three-dimensional taxonomy: valence (negative or positive), activation (deactivating or activating, also known as arousal), and object focus (retrospective outcome, concurrent activity, or prospective outcome). Research indicates that positive-activating emotions (e.g. enjoyment) should favour learning outcomes, while other types of emotions tend to hinder learning outcomes, especially negative-deactivating emotions such as hopelessness [23]. While CVT has been utilised in contemporary research in medical education [12, 24], it is unclear how widespread incorporating such frameworks into SBE research and simulation design is.

To help address the concern of whether simulation educators can consistently be deliberate in incorporating emotional experiences into SBE scenarios, a scoping review aiming to distill how simulation educators understand and study emotions was conducted. The review examined not only emotions but also closely related constructs: mood, EI, and stress.

### The peripheral constructs to emotions: mood, stress, and emotional intelligence

Previous research reports that terms such as mood and stress are closely related constructs to emotions, although they are not interchangeable terms and can be differentiated [22, 25]. EI, while not used interchangeably with emotions in the psychology or educational psychology literature, is often viewed by medical educators as one way of conceptualising emotions [11].We first identified mood as a construct of interest for our review, as we thought it may be possible for researchers to mistakenly use the term interchangeably with emotions, despite the general consensus from emotion researchers [22].

We identified stress as another construct of interest. Stress is “inextricably linked to anxiety” [26] ^(p.4)^, where such discrete negative emotions are the consequence of a stress response [25, 26]. In other words, where there is stress response, one would expect negative emotions [25]. In addition, like emotions, stress has traditionally been hard to define [27], and there was concern regarding what approach recent SBE research would take. Given the uncertainty of how stress would be conceptualised, and with stress having a close relationship with negative emotions, we reasoned that studies that examined stress held potential to infer or directly measure emotions.

We lastly considered EI. EI is deemed a highly desirable trait in medical trainees; often measured as emotional quotient (EQ) through instruments such as Bar-On’s Emotional Quotient Inventory (EQ-i) [28]. EI is associated with leadership skills, non-technical skills, reduced stress/burnout, higher job satisfaction, and better relationships with patients [29]. While EI is not an affective phenomena and moreover a trait rather than a state, it directly concerns recognising and regulating emotions [30]. Therefore, with the popularity of examining EI in SBE literature, coupled with its relationship recognising and managing emotions, we were interested in how the literature approached EI. Our interest included whether any emotional experiences would be inferred from EI measurements.

### Objectives and research questions

The objective of this scoping review was to map out how recent SBE research approached studying emotions. Our primary research question was the following: How are emotions and closely related constructs (i.e. stress, emotional intelligence, and mood) conceptualised in articles that focus on simulation environments, with medical students and trainees as the population of interest?

We formulated sets of complementary secondary questions (SQ) that align with our objective:

- (SQ1): What is known about how emotions are conceptualised?
  - ◦ (SQ1-A) Are emotions defined?
  - ◦ (SQ1-B) Do the articles cite a theory?
  - ◦ (SQ1-C) How are closely related constructs conceptualised?
- (SQ2): What is known about how emotions are measured?
  - ◦ (SQ2-A) How are other closely related constructs measured?
- (SQ3): What are the emotions medical students and trainees experienced?
- (SQ4): What kinds of emotions did medical students and trainees experience in studies that measured their stress levels?
- (SQ5): Can we infer the kinds of emotions medical trainees and students experienced in studies that measured their emotional intelligence?

## Methods

### Design

We designed and conducted our scoping review based on Arksey and O’Malley’s [31] methodological framework. We further consulted guidelines that elaborate on this framework [32], and a librarian with expertise in scoping reviews and health sciences education. Lastly, we referred to the Preferred Reporting Items for Systematic reviews and Meta-Analyses extension for Scoping Reviews (PRISMA-ScR) checklist [33]. See Appendix A for review protocol details, and Appendix B for the completed PRISMA-ScR checklist. A scoping review was chosen over a systematic review as we set out to identify the types of knowledge and research the current literature offers, and clarify key concepts—a systematic review would be better suited to assess the quality of current practices and can be a natural progression after a scoping review is conducted first [31, 34]. Our review of the literature indicates that there has yet to be a scoping review for this topic. Therefore, this knowledge synthesis work appropriately takes place before any systematic reviews.

### Stage 1: Identifying the research questions

We formulated our primary question based on our primary research objective: to map out how recent SBE research approached studying emotions. We also considered which specific aspects our review should focus on [31], namely which additional constructs related to emotions our scope would cover. There are numerous constructs that are closely related to emotions, and while they can be differentiated from emotions, they have components and features that overlap with emotions [22, 35]. Therefore, we believed there may be a range of how these terms are used and applied in research settings, warranting their inclusion.

As per our methodological framework, we refined our research question through an iterative process to balance out the vastness of our scope and the relevancy that our identified articles would yield. This included consulting our librarian and deciding to focus on SBE (versus general medical education) to enhance the review’s focus and quality. We iterated through the guidelines from Arksey and O’Malley [31] and ensured our adjusted scope aligned with our research questions and search strategy.

#### Eligibility criteria for constructs related to emotions

In terms of eligibility criteria for specific constructs, we consulted a health sciences education librarian to explore related MeSH terms and the existing literature to focus on key constructs. This allowed us to exclude less relevant terms towards our research objective such as “social stress” or terms related to clinical disorders (e.g. mood disorders).

We also decided to focus on medical students and trainees, meaning we only included studies that featured medical students, interns, residents, and fellows. If a study featured other population groups (e.g. pre-med students, nurses, attending physicians), it was only included if it featured our target population. In addition, as we were interested in the context of SBE, we sought studies that had such environments. Hence, studies that asked for general life satisfaction or a survey that asked for emotions concerning day-to-day activities were excluded. When dealing with simulations, we were mindful of whether the simulation content would be related to medical knowledge, procedure, or non-technical skills. For example, simulations of war combat via a video game would not be included in our review as the simulated content is not something we would expect a medical trainee to experience (even if there were educational components featured in the simulation).

Through the above considerations and process of exploring what our scope would be, we were able to finalise the primary research question to focus on emotions, mood, stress, and EI of medical students and trainees in a SBE context. After trials identifying the number of articles we would yield, the team agreed our scope was broad enough to adhere to our research objective.

### Stage 2: Identifying studies

We developed a search strategy with our librarian’s consultation. An iterative development process led to identifying a list of relevant keywords and MeSH terms (Table 1). Our search was carried out on *Medline* and *EMBASE* on June 22nd, 2020, exclusively looking at articles published from June 22nd, 2010 to June 22nd 2020. The initial yield of 29,329 articles decreased to 19,508 after deduplication.

**Table 1:** Search terms for scoping review

Medline	Embase
1. Resident*.tw,kf.	1. Resident*.tw,kw.
2. Residenc*.tw,kf.	2. Residenc*.tw,kw.
3. (Intern of interns*).tw,kf.	3. (Intern or Interns*).tw,kw.
4. Trainee*.tw,kf.	4. Trainee*.tw,kw.
5. Med* Student*.tw,kf.	5. Med* Student*.tw,kw.
6. Fellow*.tw,kf.	6. Fellow*.tw,kw.
7. 1 or 2 or 3 or 4 or 5 or 6	7. 1 or 2 or 3 or 4 or 5 or 6
8. Emotions/	8. Emotions/
9. Stress, Psychological/	9. Emotional Intelligence/
10. Emotional Intelligence/	10. Emotional Stress/
11. Emotion*.tw,kf.	11. Mental Stress/
12. Stress*.tw,kf	12. Emotion*.tw,kw.
13. mood*.tw,kf.	13. Stress*.tw,kw.
14. 8 or 9 or 10 or 11 or 12 or 13	14. Mood*.tw,kw.
15. 7 and 14	15. 8 or 9 or 10 or 11 or 12 or 13 or 14
16. 15 and 2010:2021.(sa_year).	16. 7 and 15
	17. 16 and 2010:2020.(sa_year).
This search yielded 11,653 hits. The search was executed on June 22nd, 2020.	This search yielded 17,676 hits. The search was executed on June 22nd, 2020.

The asterisk (*) is a truncation symbol used in the search terms to capture all possible endings of a root word, ensuring a more comprehensive search. For example, “Trainee*” will retrieve records containing “trainee”, “trainees”, “traineeship”, etc.

We chose electronic databases and decided to complement them with hand-searched articles as outlined in Arksey & O’Malley’s guidelines [31]. We chose EMBASE and Medline based on our previous experience conducting scoping reviews related to research in the fields of medical education [36]. We selected Academic Medicine and Medical Education to hand-search articles because key articles were often featured there (e.g. Artino and Pekrun [2], Pottier and colleagues [37]). Hand searching is a supplementary technique in a scoping review and not intended to be comprehensive.

### Stage 3: Study selection

For our database searches, there were three screening processes. Table 2 shows our inclusion and exclusion criteria for the title-abstract screening. Tables 3 and 4 show the criteria for the first and second full-text screening, respectively. While the first full-text screening included all medical education scenarios, the second full-text screening specifically filtered for simulation-based studies as per the iterated process outlined in Stage 1. Gaba’s definition of simulation was used to help screen the articles, where simulation was defined as “a technique—not a technology—to replace or amplify real experiences with guided experiences that evoke or replicate substantial aspects of the real world in a fully interactive manner” [38] (p.i2). We referred to Gaba’s article that describes various dimensions of simulations to operationalise different criteria and types of simulators.

**Table 2:** Inclusion and exclusion criteria for title-abstract screening

Inclusion criteria	Exclusion criteria
Empirical study, reviews, conference proceedings, commentaries, editorials, conference papers	Magazine articles, one-off diagrams, supplementary articles, theses, dissertations, abstracts
Medical students, interns, residents, and fellows (if any of these are included)	Nurses, dentistry students, paramedics, physicians, doctors, patients (if only these are exclusively included)
Emotions, mood, stress, and emotional intelligence	Depression, burnout, attitudes.

**Table 3:** Inclusion and exclusion criteria for the first full-text screening

Inclusion criteria	Exclusion criteria
English articles	Non-English articles
Empirical studies	Review, editorial, opinion pieces, conference abstracts, conference listings, authors’ response, letter to the editor
Medical students, interns, residents, and fellows (if any of these are included)	Nurses, dentistry students, paramedics, physicians, doctors, patients (if only these are exclusively included
Emotions, emotion regulation, discrete emotions (e.g. anxiety, anger, sadness)	Attitudes, personality
Mood	Depression, post-traumatic stress disorder, mood disorders
Stress, stressors, distress, stress coping (regulation), physiological indicators of stress	Burnout, emotional exhaustion, depersonalisation, empathy (if only these are exclusively included)
Emotional intelligence

**Table 4:** Inclusion and exclusion criteria for the second full-text screening

Inclusion criteria	Exclusion criteria
There is at least one simulator mentioned: cadavers, mannequins, screen-based simulators, VR/AR simulator, role playing, standardised patients, task trainer.	There are no simulators mentioned
Objective Structured Clinical Examination (OSCE), Fundamentals of Laparoscopic Surgery (FLS) training, “cases”	Non-medical simulations, such as computer simulations (e.g. simulations of data), flight simulations, computer games (that are not focused on medical-related tasks)
Participants: Medical students, interns, residents, and fellows (if any of these are included)	Nurses, dentistry students, paramedics, physicians, doctors, patients (if only these are exclusively included

Each of the screening processes involved a team of reviewers (six for title-abstract screening, four for both full-text screenings), where each article was screened by a pair. A pilot preceded every screening process to ensure 75+% interrater reliability before proceeding [39]. Re-calibrations took place after 1/3 and 2/3 of the articles were screened.

**Fig. 1:**
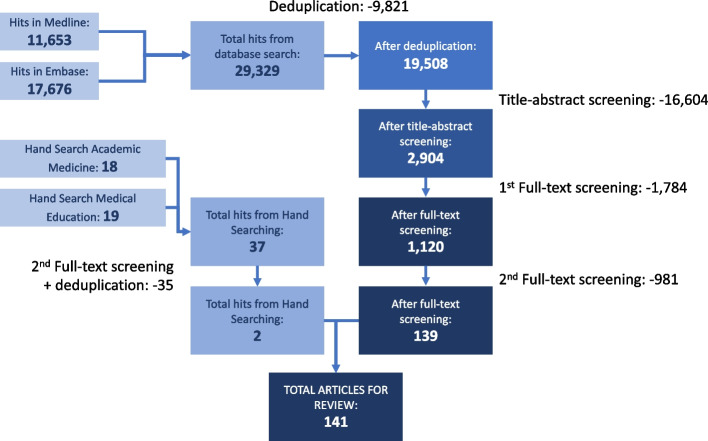
Flow diagram of study selection process

In addition to searching electronic databases, screening of hand-searched articles involved four reviewers. These articles (37) were screened for SBE content, similarly to our full-text screenings. After deduplication, two articles were added (please see Fig. 1 for the flow diagram).

### Stage 4: Data charting and reporting

We finalised our data extraction sheet and strategy through consultation of multiple resources, our librarian, and pilot testing. Two reviewers were chosen in the end to extract the articles to ensure high interrater reliability. See Appendix C for details.

The final data extraction sheet included three categories of data: (1) information pertaining to the publication of the article (e.g. author, year); (2) information about the constructs of interest (e.g. whether the article focused on emotions or closely related constructs, what measurements were used); and (3) information on the simulation based on Gaba’s dimensions of simulation applications [38].

### Stage 5: Collating, summarising, and reporting the results

We analysed our data descriptively; we summarised general characteristics of the papers through various charts to provide a broad overview of study characteristics. We report our summary to answer our research questions while providing context.

## Results

The database search (139) combined with hand searching (2) yielded a total of 141 articles that met the inclusion criteria for review [194–334].

### Study characteristics

Our results showed that research in emotions and related constructs have been increasing over the years, with most of the articles published in western countries (e.g. USA, UK). The vast majority (83.0%; 117 articles) were quantitative studies, while the bulk of the populations featured were medical students (56.7%; 80 articles) and residents (43.4%; 61 articles).

Stress was featured the most in the articles we identified (62.4%; 88 articles), followed by emotions (31.9%; 45 articles) and EI (24.1%; 34 articles). Only 1 article (0.7%) featured mood. Appendix D contains more details about study characteristics, including simulation characteristics.

### Conceptualisation, measurement, and experiences of emotions

Our results indicated that most of the articles that focused on emotions did not formally define emotions: 35 (77.8%) of the 45 articles on emotions [40–73]. Seven (15.6%) articles [9, 74–79] defined a type or a discrete emotion (e.g. anxiety), 2 [80, 81] (4.4%) provided formal definitions, while 1 [82] defined both a formal definition for the term emotion, and separate definitions for discrete emotions (e.g. anxiety). For specific types of emotions, achievement emotions were the only type identified (as opposed to other types such as epistemic emotions—emotions that relate to knowledge and generation of knowledge [83]). Definitions of discrete emotions focused mainly on negatively valenced emotions such as anxiety and embarrassment. Amongst the articles that formally defined emotions, the circumplex model of emotion was referenced once and CVT was referenced four times.

**Fig. 2:**
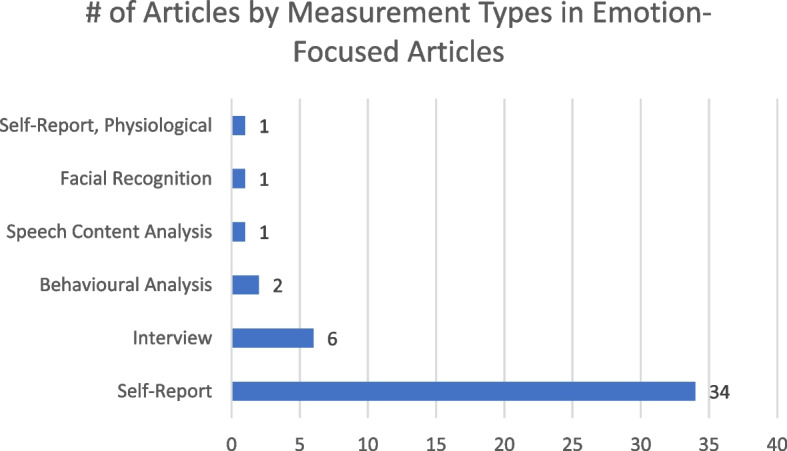
Number of articles by measurement types for emotions-focused articles

**Fig. 3:**
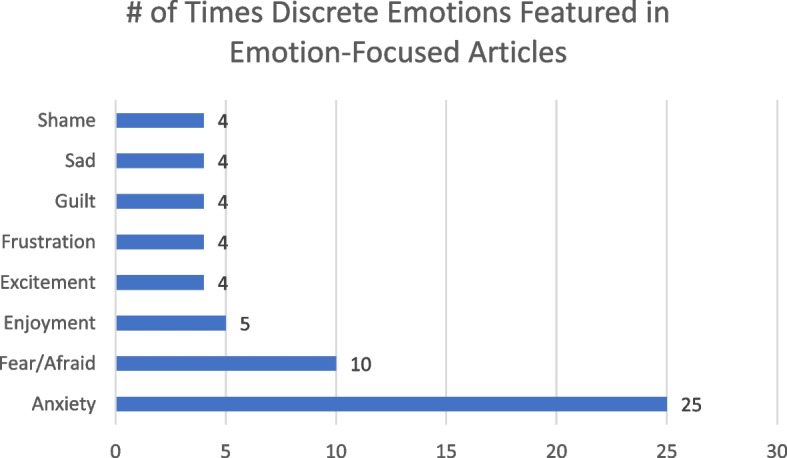
Number of appearances for discrete emotions

Figure 2 shows that self-report measures (34 articles) were the most common method of collecting data on emotions. Besides custom self-made instruments [43, 44, 47, 50, 55, 56, 63, 69, 74, 84], the State-Trait Anxiety Inventory [40, 41, 60, 64–67, 79] (STAI; or a variation of it) was the most employed instrument (featured 8 times in emotion-focused articles). The next most common instruments were the Achievement Emotions Questionnaire [74, 77, 82] (AEQ), and a scale based on Barrett and Russel’s work on emotions [48, 53, 76] (i.e. the circumplex model [85]), each featured 3 times. See Appendix E for more details on the wide array of other instruments used in studies. Figure 3 shows that anxiety and fear (captured in 25 [9, 40, 41, 44, 46, 47, 50, 52, 55, 59, 60, 62, 64–67, 69, 73, 74, 77–81, 84] and 10 articles [44–47, 50, 52, 55, 57, 59, 64], respectively) were the most commonly measured emotions across the 45 articles that focused on emotions. Few positive emotions were captured in the studies, with excitement and enjoyment being the most frequently measured ones, but only being mentioned in four [50, 53, 56, 80] and five articles [53, 57, 71, 74, 77], respectively.

### Conceptualisations and measurements of other constructs

Sixty-four of the 88 articles that focused on stress (72.4%) did not formally define stress with an explicit reference to a theoretical framework. Sixteen (18.2%) articles [37, 65, 66, 72, 79, 86–96] did, however, formally define what stress was, while 6 articles [59, 97–101] (6.8%) defined related terms to stress such as distress and stressors. Lastly, 2 articles [102, 103] (2.3%) defined both stress and related terms. For the 16 papers that formally defined stress, 8 [65, 66, 72, 79, 86, 92–94] relied on conceptualisation of stress stemming from a physiological-oriented (physiogenic) approach (e.g. Selhye’s General Adaptation Syndrome [104]), while 6 of the papers [37, 66, 90, 91, 95, 96] from a psychological-oriented (psychogenic) approach (i.e. Lazarus’ Transactional Model [105]). Overall, 3 papers [79, 87, 102] relied on definitions that drew from multiple approaches. It should be noted that 1 paper that explicitly defined stress did not provide any references (however took a physiological-oriented approach to defining stress) [88].

**Fig. 4:**
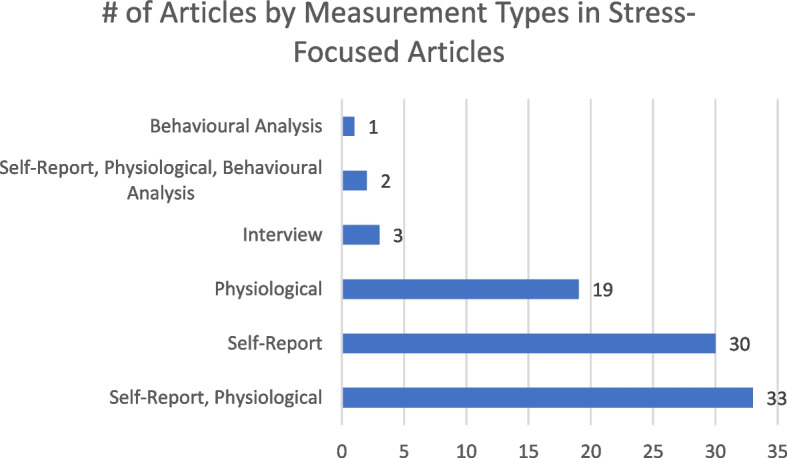
Number of articles by measurement types for stress-focused articles

Figure 4 shows the measurements that stress articles employed. Studies using both self-reports and physiological measures were the most common (33 articles [37, 65–67, 72, 73, 87, 90, 91, 94, 96, 98, 101, 106–125]; 37.5%), followed by articles solely relying on either self-reports alone (30 articles [56, 60, 63, 64, 68, 70, 71, 84, 92, 95, 99, 102, 103, 126–142]; 34.1%) or physiological measures alone (19 articles [55, 62, 69, 79, 86, 88, 89, 93, 143–153]; 21.6%). There were two articles [100, 154] (2.3%) that used behavioural analysis in addition to self-reports and physiological measures. Heart rate or heart rate variability-related instruments were the most common (32 articles [65, 67, 69, 79, 86–90, 94, 96, 98, 100, 106–111, 113, 114, 117–120, 124, 143, 145, 148, 151, 152, 154]; 36.4%) for physiological measures. For self-report measures, the STAI was the most common (20 articles [37, 64, 67, 72, 90, 94, 98, 100, 103, 106, 108–110, 113, 114, 116, 118, 123, 124]; 22.7%).

Nine EI articles [7, 9, 98, 155–160] (26.5%) formally defined EI. Thirteen articles [57, 60, 97, 161–170] (38.2%) defined a construct directly related to, or a subordinate construct of EI (e.g. empathy, emotional skills). The most common measurement in these articles was the Jefferson Scale of Physician Empathy [171] (JSE; 6 articles [162, 166, 167, 169, 170, 172]; 17.6%), followed by The Trait Emotional Intelligence Questionnaire [173] (TEIQue; 4 articles [98, 158, 165, 174]; 11.8%) and The Mayer-Salovey-Caruso Emotional Intelligence Test [175] (MSCEIT; 3 articles [9, 159, 160]; 8.8%). The EI articles exclusively focused on EI did not infer emotions from their measures.

The one article that focused on mood [176] utilised the visual analogue scales (VAS) from Bond and Lader [177] to assess “high” (positive) and “low” (negative) mood, and hence did not measure any discrete mood or emotions. It did not formally define mood or emotions.

## Discussion

### Primary question

Our primary research question was: “How are emotions and closely related constructs (i.e. stress, emotional intelligence, and mood) conceptualised in articles that focus on simulation environments, with medical students and trainees as the population of interest?” Based on our results, the short answer is that the SBE literature concerning emotions and related constructs tend to be light on theory, relying on previous research findings to orient their research. The literature had an affinity towards focusing on negative emotions (e.g. anxiety) and made connections between negative emotions and stress. Our review also further acknowledged the importance of these psychological constructs in education—hence acknowledging the desirability of EI in trainees (although no emotions are inferred from students’ EI levels). The more elaborate version of our answer was obtained through answering the secondary questions we proposed.

### Secondary questions

#### Conceptualisation and measurements of emotions

SBE literature often discusses emotions informally, without explicit definitions or references. This was evident as most emotion-focused articles (35/45) did not provide a definition for emotions. We believe this may partially be due to the studies’ scopes and intentions, which are shaped by norms and trends in SBE. While a definition by itself is not a theory, it serves as an important part of a psychological theory. As these articles did not formally cite any theories either, we concluded that they lacked a theoretical-based conceptualisation of emotions. Based on our results, we believe that recent work in the SBE literature resembles the early emotion research activities in the realm of traditional education research in that it focuses on specific discrete negative-activating emotions and relies on self-report measures [178].

Many studies concentrated on negative emotions. For example, Kim’s study [74] looked at how medical students’ anxiety and boredom in class were associated to levels of anxiety in Objective Structured Clinical Examinations (OSCEs). Fraser and colleagues’ [48] focused on negative emotions elicited from simulated patient death. It was therefore not surprising that measurements towards emotions largely included self-reports (34 articles of 45 that focused on emotions) that focused on negative emotions such as anxiety (8 articles using STAI [179] to measure this) and other negative emotions such as fear (measured via the Fear of Negative Evaluation Scale [180]) and shame (measured via Experimental Shame Scale [181]). We noted that studies that did not rely on self-reports also followed the trend of identifying negative emotions in SBE [45, 52, 58, 59].

The emphasis on negative emotions is understandable. This is especially so when we consider how medical trainees face training scenarios that are *meant* to elicit negative emotions, due to the nature of the topic the scenarios deal with, and the pressure for achieving high performance. For example, articles such as Groot’s article [58] featured medical students taking on residency-level simulations that featured emergency room acute care cases (e.g. anaphylaxis, acute myocardial infarction). These simulations placed the students in an emotionally charged situation where negative emotions such as anxiety (i.e. stress) would be elicited, in part, due to the advanced and challenging nature of the medical issues they needed to manage. Students strived to perform well but ultimately reported stress and disappointment in themselves failing to meet their own expectations. Our other reviewed articles follow this example in terms of dealing with emotionally charged topics and scenarios likely to elicit negative emotions. Bloomfield and others’ article [51] featured students communicating with dying patients and their family members. Bauer and others’ article [79] had residents for their first time be given a high-fidelity mannikin in a scenario where the simulated patient was intubated during an intra-hospital transportation, but had oxygen desaturation, and was under mechanical ventilation. Summing up these examples, we report that the typical SBE scenario in our review featured stress-inducing, negative emotion eliciting experiences—explaining why the literature perhaps tends to focus on negative emotions.

Our results showed that four articles referenced the CVT to define emotions, with three using related measures (i.e. Achievement Emotion Questionnaire; AEQ [182]). While focusing on specific emotions such as anxiety has merit, extending the acknowledgement of emotions’ role beyond a specific discrete emotion such as anxiety, or a type of emotion would benefit the SBE literature by providing a more comprehensive picture of the potential role other emotions play. Broad examples of this include studying emotions during simulation versus after simulation (debriefing); effects of positive-activating emotions (enjoyment) versus positive-deactivating emotions (relief) and so on. We make a positive note that CVT—one of the most suitable frameworks for being applied to SBE research—was cited by several of the few articles that *did* rely on theory. This suggests that SBE researchers are on the right track in conceptualising emotions in academic contexts.

While we highlighted the need to look beyond negative emotions in SBE research, studies such as Butteris and colleague’s [50] illuminates the rationale for focusing on negative emotions. Unlike the rule of thumb that negative emotions are generally undesirable in education, the study’s facilitators’ consensus seemed to be that completing a simulation scenario involving a neonatal death or caring for a simulated HIV-positive toddler requires trainees to experience negative emotions so that they are motivated to reflect on their competency and preparedness [50]. We note that the study emphasised post-simulation debriefings to help trainees adaptively digest these negative emotions. Though the non-profession-specific educational (e.g. high school, higher education) emotion literature acknowledges that emotions such as anger may be beneficial in specific contexts [23, 183], the contexts featured are difficult to compare to what medical students and trainees experience.

#### Conceptualisations and measurements of related constructs to emotions

Like emotions, most articles focusing on stress (64/88) did not offer formal definitions. Instead, they referenced previous work and expert opinions to establish research directions. Further, papers featuring stress tended to simply conceptualise stress from a biological, physiological approach. The assumption may be that readers of medical education journals do not expect formal explanations of stress, as they are familiar with the biological components of stress responses (e.g. activation of the hypothalamic-pituitary-adrenal and the sympathetic-adrenal-medullary axes). Hence, many authors simply mention this physiological side of stress in lieu of citing a theoretical framework. We note that authors who measured constructs such as anxiety as an indicator of stress were measuring a construct that is different from stress, albeit related [184].

Measurements of stress-related articles were similar to emotion-related articles in that they often employed instruments such as the STAI [179]. In other words, articles that focused on stress, while not studying a wide variety of emotions, still often examined negative-activating emotions such as anxiety. This is supported by our findings that of the 18 articles that examined both emotions and stress, 16 featured STAI for measuring anxiety. What was different from emotion-related articles, however, was the frequent reliance on multiple channels of data: 36.4% of stress articles included both self-report and physiological measures to infer stress as opposed to just 2.1% of the emotion articles. We believe this is a symptom of the SBE literature not embracing formal definitions of emotions. Formal definitions of emotions will tend to agree that emotions are multi-componential psychological responses which include a combination of affective, cognitive, physiological, motivational, and expressive processes [22]. Therefore, measuring emotions should go beyond self-report measures and should also measure the physiological (e.g. skin conductance, heart rates) and expressive (e.g. facial expressions, speech) aspects of emotions.

The conceptualisation of EI was more formal relative to emotions and stress (26.5% of the articles with formal definitions versus 4.4% and 18.2% of the articles with formal definitions for emotions and stress respectively). Articles often cited ideas related to Goleman [185] and Mayer [186] (theories claiming EI as a type of intelligence), in addition to citing Petrides [187] in reference to the theory of trait emotional intelligence. If we consider that 13 articles that did not define EI still defined the construct they were measuring (e.g. empathy), 64.7% of EI articles featured formal definitions. This finding seems to signal a trend in SBE where constructs such as emotions are just emerging and therefore lack theoretical integration, while emotional intelligence may be a more established topic with a more matured approach.

We lastly note that we only identified one article that focused on mood. We report that the SBE literature does not seem to interchangeably use the term mood and emotions.

#### Emotions of medical students and trainees

We report that anxiety and fear were the most captured emotions in our reviewed articles. According to CVT’s classification, both are negative-activating emotions, indicating their similarities. The captured emotions reflected how the chosen instruments aligned with the studies’ objectives of investigating negative-activating emotions. In other words, if the study sought to investigate anxiety in medical students, the emotional profile reported will mainly be anxiety. Overall, as discussed earlier, we report that while students and trainees do experience positive emotions (e.g. excitement, enjoyment) during certain simulation scenarios, the literature in our review more often captured negative emotions (e.g. anxiety, fear, frustration, guilt) due to the intensity of the scenarios (e.g. simulated patient death [48]), and the high expectations set for learners. According to the CVT, high expectations means high appraisal of value (i.e. learners perceive their performance in a simulation to be important), which lead to emotions with high levels of intensity [188]. This is especially the case when one’s control over a situation is uncertain or low (i.e. the difficulty of the simulation is high, or there are uncontrollable factors in a simulation) [188].

#### Inferring emotions from stress and emotional intelligence

Studies that focused on both stress and emotions (predominantly anxiety) tended to infer anxiety from stress levels of their participants. It is also interesting to note that two of the studies [58, 61] that focused on emotion reported “stress” as a type of emotion their participants experienced. Overall, SBE articles tended to acknowledge that anxiety is an expressive component of stress. However, we think caution is warranted in using the term stress, emotions, and anxiety interchangeably. Anxiety is just one of many different stress responses [189], and hence sole reliance on measurement of anxiety may be limited as opposed to relying on multiple measures that also take into account physiological measures or behavioural coding.

Further, when examining what other discrete emotions educators could intend on introducing and measuring in SBE, considering that the CVT illustrates how interchanging stress is with the term emotion, this interchangeability may potentially lead to a narrow capture of emotions. While anxiety is a negative-activating emotion like anger and shame, they are not identical and have different implications for learning [2, 190]. Therefore, measuring stress may capture whether one is feeling anxious or not, but not adequately capture anger or shame. For other emotions that are still negative but are deactivating (e.g. boredom, hopelessness, sadness, disappointment), this issue becomes much more prominent. Finally, measuring stress would not capture positive emotions, missing emotions such as enjoyment and curiosity.

While McNaughton’s discourse analysis revealed that educators can view emotions as a skillset, we believe, from an educational psychology perspective, that there is a distinction between having the ability to understand one’s own and others’ emotions (i.e. EI) [29, 186] and the actual experience of feeling specific emotions. Aligned with this, our results showed that the researchers that focused on EI did not infer emotions from EI measurements. The closest inference would be Dohms and others [191] reporting that students with higher empathy will have better emotion regulation, leading to a calmer emotional profile in stressful situations relative to their peers. We therefore presume that SBE researchers do not confuse experiencing specific discrete emotions with levels of emotional intelligence.

Specific calls to action based on our research partially echoes Leblanc and Posner’s review [10]: simulation designers and researchers should ask what emotions they are deliberately or potentially introducing to the participants and consider the impact they may have. However, in thinking and conceptualising this, we would like to extend their call by specifying *how* to be deliberate and consider integrating a theoretical framework that can formally define what emotions are.

### Contextual factors and future directions

When interpreting our study, certain contextual factors matter. Our results show that SBE literature is prominent in the western world, with USA, UK, Germany, Canada, and France comprising 66.6% of the articles. The role of culture may influence studies [192, 193] and increasing cultural diversity in SBE emotions research is therefore an important future direction. Specifically examining SBEs offered in institutions in various nations is one example of this direction. Further, our results indicate that certain types of simulation structures and contexts were underrepresented. For example, only 6.4% of our studies featured interprofessional teams, while certain simulators such as augmented reality or virtual reality simulators were featured much less (3.5%). Focusing the investigation of emotions in such specific SBE contexts could be valuable future directions.

We note limitations of our scoping review process, including our limited selection of electronic databases, not drawing on more than two journals for hand searching, especially from journals that are SBE-focused. Other limitations include our review focusing on just empirical articles, lacking additional screenings of identified articles’ reference lists, and the lack of consulting content experts. In addition, as our study inclusion criteria was quite broad (e.g. including individual-based, team-based simulations, and a wide range of simulators), our findings may have different applications when focused on specific types of SBE. While our results are applicable to the general landscape of the SBE literature, extrapolating our findings to specific subfields of SBE may warrant care.

Our study had some notable strengths as well. Other than the expected standards of following a scoping review framework and being consulted on our search decisions by an expert librarian, we have also conducted screenings, data extraction, and analysis via evidence-based practices. From identifying the affect-related constructs to be studied, to how we conceptualised simulations and their different features, decisions were based on prominent ideas to ensure consistent and accurate intake and analysis of data. Our appendix and body of the manuscript also offer full transparency in all the steps we have taken based on Arksey and O’Malley’s framework.

Future studies could focus on exploring more databases with additional consultation from content experts. Further, our study only extends to the middle of 2020. At the time we wrote this article, the COVID-19 pandemic had left its impact on healthcare education worldwide; it would be interesting to see whether there are any shifts in directions and activities within the SBE literature. Other future directions include this review being a basis for a systematic review on how theoretical frameworks guide emotion-focused SBE research and the interpretation of the results.

## Conclusion

We presented a scoping review that aimed to describe the current state of SBE literature pertaining to the conceptualisation of emotions and related constructs: stress, emotional intelligence, and mood. Our results revealed that authors of SBE tend to omit including a theoretical framework for conceptualising emotion-related constructs in their study. We also highlight another tendency amongst SBE studies: capturing negative emotions such as anxiety, where studies that examine stress also often evaluate the levels of anxiety of learners. This tendency of capturing negative emotions reveals that, at first glance, medical trainees and students are stricken with anxiety, fear, and guilt. However, we note that this finding partially stems from studies setting out to measure negative emotions that are also identified as *important* in medical education. Unlike in more traditional fields of education (i.e. Kindergarden-12, Higher Ed), negative emotions seem to have a more profound and authentic role in facilitating learning in SBE. For example, Butteris and colleagues identified how negative emotions such as frustration and helplessness facilitated motivation for preparedness [50]. Future research that embraces theoretical frameworks such as CVT should equip researchers with the tools they need to critically interpret the impact of such emotions in SBE. In addition, synthesis work focusing on specific types of SBE environments in relation to emotion-related constructs (e.g. interprofessional SBE using virtual reality simulators), and the role of emotion regulation [21] supporting instructional design can serve as next steps. This line of work can illuminate the roles of emotions in SBE and how to best support students’ experiences of these influential psychological states that are associated with learning, performance, and psychological well-being.

### Supplementary Information

**Additional file 1: Appendix A.** Review Protocol. **Appendix B.** PRISMA Extension for Scoping Reviews (PRISMA-ScR) Checklist. **Appendix C.** Data Charting Process. **Appendix D.** Study characteristics. Figure D1. Articles published by year. Figure D2. Percentage of first (or corresponding) authors’ affiliated institution’s country. Figure D3. Number of articles by specific study design. Figure D4. Number of articles featuring population type. Figure D5. Number of times mood, EI, stress, or emotions featured in the articles. Figure D6. Percentage of articles by combination of constructs featured. Table D1. Types of simulators featured. **Appendix E.** List of scales featured for measuring emotions.

## Data Availability

The list of articles included in this review can be found in the reference section. The keywords and search strategy we have used is outlined in the “ Methods” section of this article and further details can be found in the Appendix.

## Abbreviations

SBE: Simulation-based education
EI: Emotional intelligence
CVT: Control Value Theory of Achievement Emotions
