# Supplementary material for: A scoping review of emotions and related constructs in simulation-based education research articles
Source: Adv Simul (Lond). 2023 Sep 16;8:22. doi: 10.1186/s41077-023-00258-z (PMC10505334; doi:10.1186/s41077-023-00258-z)
Supplement: Supplementary file 1 — Additional file 1: Appendix A. Review Protocol. Appendix B. PRISMA Extension for Scoping Reviews (PRISMA-ScR) Checklist. Appendix C. Data Charting Process. Appendix D. Study characteristics. Figure D1. Articles published by year. Figure D2. Percentage of first (or corresponding) authors’ affiliated institution’s country. Figure D3. Number of articles by specific study design. Figure D4. Number of articles featuring population type. Figure D5. Number of times mood, EI, stress, or emotions featured in the articles. Figure D6. Percentage of articles by combination of constructs featured. Table D1. Types of simulators featured. Appendix E. List of scales featured for measuring emotions. [file 41077_2023_258_MOESM1_ESM.docx]

# Appendix A

## Review Protocol

Below is the general protocol we followed for our scoping review, based on Arksey and O’Malley’s framework^1^ for conducting scoping reviews. We did not register our protocol.

##
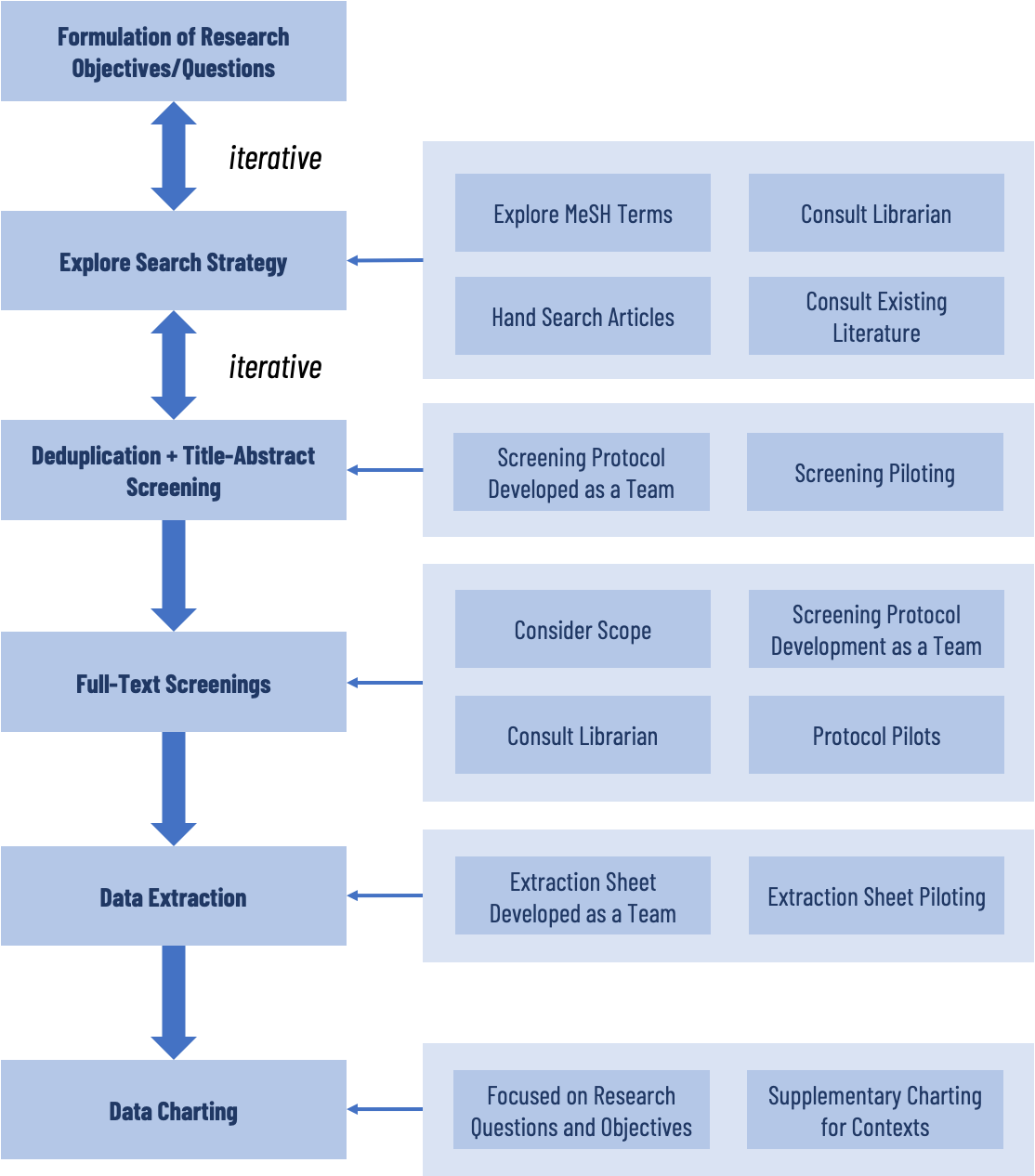


# Appendix B

## PRISMA Extension for Scoping Reviews (PRISMA-ScR) Checklist

| **Section** | **Item** | **PRISMA-ScR Checklist Item** | **Check Results** |
| --- | --- | --- | --- |
| **Title** | 1 | Identify the report as a scoping review. | Yes (Manuscript) |
| **Abstract** |  |  |  |
| Structured Summary | 2 | Provide a structured summary that includes (as applicable) background, objectives, eligibility criteria, sources of evidence, charting methods, results, and conclusions that relate to the review questions and objectives. | Yes (Manuscript) |
| **Introduction** |  |  |  |
| Rationale | 3 | Describe the rationale for the review in the context of what is already known. Explain why the review questions/objectives lend themselves to a scoping review approach. | Yes (Manuscript) |
| Objectives | 4 | Provide an explicit statement of the questions and objectives being addressed with reference to their key elements (e.g., population or participants, concepts, and context) or other relevant key elements used to conceptualize the review questions and/or objectives. | Yes (Manuscript) |
| **Methods** |  |  |  |
| Protocol and registration | 5 | Indicate whether a review protocol exists; state if and where it can be accessed (e.g., a Web address); and if available, provide registration information, including the registration number. | Yes (Appendix A) |
| Eligibility criteria | 6 | Specify characteristics of the sources of evidence used as eligibility criteria (e.g., years considered, language, and publication status), and provide a rationale. | Yes (Manuscript) |
| Information sources | 7 | Describe all information sources in the search (e.g., databases with dates of coverage and contact with authors to identify additional sources), as well as the date the most recent search was executed. | Yes (Manuscript) |
| Search | 8 | Present the full electronic search strategy for at least 1 database, including any limits used, such that it could be repeated. | Yes (Manuscript) |
| Selection of sources of evidence | 9 | State the process for selecting sources of evidence (i.e., screening and eligibility) included in the scoping review. | Yes (Manuscript) |
| Data charting process | 10 | Describe the methods of charting data from the included sources of evidence (e.g., calibrated forms or forms that have been tested by the team before their use, and whether data charting was done independently or in duplicate) and any processes for obtaining and confirming data from investigators. | Yes (Appendix C) |
| Data items | 11 | List and define all variables for which data were sought and any assumptions and simplifications made. | Yes (Appendix D) |
| Critical appraisal of individual sources of evidence | 12 | If done, provide a rationale for conducting a critical appraisal of included sources of evidence; describe the methods used and how this information was used in any data synthesis (if appropriate). | Not applicable |
| Summary measures | 13 | Not applicable for scoping reviews. | Not applicable |
| Synthesis of results | 14 | Describe the methods of handling and summarizing the data that were charted. | Yes (Manuscript) |
| Risk of bias across studies | 15 | Not applicable for scoping reviews. | Not applicable |
| Additional analyses | 16 | Not applicable for scoping reviews. | Not applicable |
| **Results** |  |  |  |
| Selection of sources of evidence | 17 | Give numbers of sources of evidence screened, assessed for eligibility, and included in the review, with reasons for exclusions at each stage, ideally using a flow diagram. | Yes (Manuscript) |
| Characteristics of sources of evidence | 18 | For each source of evidence, present characteristics for which data were charted and provide the citations. | Yes (Manuscript). However, due to the scope of the review, not all evidence were examined thoroughly in detail in the main manuscript |
| Critical appraisal within sources of evidence | 19 | If done, present data on critical appraisal of included sources of evidence (see item 12). | Not applicable |
| Results of individual sources of evidence | 20 | For each included source of evidence, present the relevant data that were charted that relate to the review questions and objectives. | Yes (Manuscript) However, same caution from item 18 applies here. |
| Synthesis of results | 21 | Summarize and/or present the charting results as they relate to the review questions and objectives. | Yes (Manuscript) |
| Risk of bias across studies | 22 | Not applicable for scoping reviews. | Not applicable |
| Additional analyses | 23 | Not applicable for scoping reviews. | Not applicable |
| **Discussion** |  |  |  |
| Summary of evidence | 24 | Summarize the main results (including an overview of concepts, themes, and types of evidence available), link to the review questions and objectives, and consider the relevance to key groups. | Yes (Manuscript) |
| Limitations | 25 | Discuss the limitations of the scoping review process. | Yes (Manuscript) |
| Conclusions | 26 | Provide a general interpretation of the results with respect to the review questions and objectives, as well as potential implications and/or next steps. | Yes (Manuscript) |
| **Funding** | 27 | Describe sources of funding for the included sources of evidence, as well as sources of funding for the scoping review. Describe the role of the funders of the scoping review. | Not applicable (no funding) |

# Appendix C

## Data Charting Process

We have developed a data extraction sheet so that we could systematically extract data for charting. We ran pilots of our data extraction sheet, where the first three pilots included six reviewers. However, when it came to extracting data that were more inference-based (e.g., deciding what construct the article focused on) we realized that our interrater reliability was below the cut-off point of 75%. We therefore decided to continue collecting feedback from our reviewers, and refine our extraction sheet, while we also narrowed down the number of our reviewers. Our fifth and final pilot included four reviewers. BA (the first author of this paper) was the lead reviewer for this project, and so we decided to compare others’ data extraction results to BA’s and calculate the number of Excel cells that significantly differed from his extraction. Reviewers CL, EB, and MM yielded agreement ratings of 88.81%, 91.53%, and 95.59% with BA. We in the end decided that MM and BA would extract the data from the articles.

# Appendix D

## Study Characteristics

Below is a figure (Figure D1) that shows the articles published by year and illustrates research in emotions and related constructs have been increasing over recent years.

**Figure D1.** Articles Published by Year

Figure D2 shows the percentage of first/corresponding authors’ affiliated institution’s country. The United States (30.5%; 43 articles), United Kingdom (9.9%; 14 articles), and Germany (9.9%; 14 articles) were the most common countries affiliated with the first author.

**Figure D2.** Percentage of First (or Corresponding) Authors’ Affiliated Institution’s Country

Our results also showed that the vast majority (83.0%; 117 articles) were quantitative studies, while only 5.0% (7 articles) and 12.1% (17 articles) were qualitative and mixed/multi-method studies, respectively. Figure D3 illustrates articles featuring specific study designs. It should be noted that an article may feature multiple specific study designs simultaneously (e.g., have a focus group interview, along with a group comparison design). While 34 studies (24.1%) featured a randomized controlled trial (RCT) design, the single group design (53.9%; 76 articles) was the most employed design.

**Figure D3.** Number of articles by specific study design

For interpreting data from Figure D2 and D3, it should be noted that there were two types of categorizations we have made with the articles in our review when it came to study design: broad and specific. The former focused on categorizing articles based on whether they applied quantitative, qualitative, or mixed/multi-method analyses. We acknowledge that there is a distinction between mixed and multi-method^2^, but have opted to group them up together as doing so still gives us a clear picture concerning whether the literature was dominated by quantitative methods.

The latter categorization aimed to delve into the specific types of study design. While we did start out with various terms guided by work from Turner and others^3^, these terms were eventually replaced by the terms produced through the iterative refinement process from our pilot phases; these were based on some of the co-authors’ experience conducting scoping reviews^4^: 1) Single Group: If all the participants experienced the same training/education; 2) Group Comparison: If there are two or more groups and are being compared to each other; 3) RCT (Randomized Controlled Trials): If there is randomization and a control group for comparison (regardless of blinding). This includes “single group” studies if participants were randomly assigned to specific sub groups and counterbalanced for comparison (i.e.,, randomized crossover study), despite the fact that, technically, the students were experiencing the same training in the end; 4) Survey Study: if the study solely relies on a survey sent out to students to poll data, without featuring any intervention or educational activity in their study itself (e.g., ask about a simulation experience but not have the students go through the simulation); and 5) Interview: Studies that involve interviewing. This included studies that featured focus groups. With these terms our reviewers all agreed upon, we were able to conduct the data extraction consistently.

Figure D4 shows the type of relevant populations featured in the articles (again, studies can feature multiple types simultaneously). Medical students (56.7%; 80 articles) and residents (43.3%; 61 articles) made up the bulk of the populations featured. Interns and fellows only represented 3 (2.1%) and 5 articles (3.5%), respectively. We further note that 18 articles (12.8%) featured populations that were not relevant to our scope (e.g., physicians, nursing students).

**Figure D4.** Number of articles featuring population type

Figures D5 and D6 exhibit the number of articles featuring the constructs our scope sought to capture. Stress was the construct that was featured the most (62.4%; 88 articles), followed by emotions (31.9%; 45 articles), and emotional intelligence (24.1%; 34 articles). There was only 1 article (0.7%) that featured mood. As figure 7 shows, we note that there were only 2 articles (1.4%) that examined emotions, stress, *and* emotional intelligence; 3 articles (2.1%) that looked at both stress and emotional intelligence, and 2 articles (1.4%) that looked at both emotions and emotional intelligence. There were 18 articles that looked at both emotions and stress (12.8%).

**Figure D5.** Number of times Mood, EI, Stress, or Emotions Featured in the Articles

**Figure D6.** Percentage of Articles by Combination of Constructs Featured

Regarding simulation characteristics, we identified 90 (63.8%) articles that featured simulations done individually, with 49 (34.8%) featuring single-profession teams, and 9 (6.4%) featuring interprofessional teams. We also identified 35 (24.8%) articles that focused on both technical and non-technical skills, 63 articles (45.4%) that focused on just technical skills, and 36 articles (25.5%) that focused on just non-technical skills. For the type of simulators, we found that use of standardized participants (49 articles; 34.8%) and mannequins (48 articles; 34.0%) were the most common. See Table D1 below.

**Table D1.** Types of Simulators Featured

| **Simulator Type** | **Count** |  | **%** |
| --- | --- | --- | --- |
| Role Playing | 28 |  | 19.9% |
| Standardized Participants (SPs) | 49 |  | 34.8% |
| Task Trainer | 26 |  | 18.4% |
| Screen-Based | 9 |  | 6.4% |
| AR/VR Based (with head-sets) | 5 |  | 3.5% |
| Mannequin | 48 |  | 34.0% |
| Human Cadavers | 12 |  | 8.5% |
| Animal Cadavers | 1 |  | 0.7% |
| Live Animals | 1 |  | 0.7% |

We note that we differentiated the use of the Role Playing and Standardized Participants (SPs). We counted the article towards former if the simulation featured participants (not SPs) role playing (e.g., a medical student role playing as a family member of the patient). Therefore, even though SPs are technically role-playing, if the simulation did not have participants role-playing, SP articles did not count towards role playing.

Further, while full-body mannequins were counted towards the mannequin category in our review, if it was just a part of a mannequin (e.g., a limb of a mannequin), we considered it to be a task trainer, not a mannequin. Our term Screen-Based simulator only accounted for simulators that had digital interfaces on a traditional flat screen (e.g., desktop monitors, laptops, TV screens, etc.). Augmented reality and virtual reality (AR/VR) simulations were only counted if they were utilized through a headset.

# Appendix E

## List of Scales Featured for Measuring Emotions

| **Instrument Name** | **# of Times Featured** |
| --- | --- |
| Custom Instrument | 11 |
| State-Trait Anxiety Inventory (STAI) | 8 |
| Interview: Thematic Analysis | 3 |
| Barrett and Russell's Semantic Structure of Emotion Scale | 3 |
| Achievement Emotions Questionnaire (AEQ) | 3 |
| 17-item Training Evaluation Inventory (TEI) | 1 |
| NASA task load | 1 |
| Generalized Anxiety Disorder Scale (GAD-7) | 1 |
| Experiences in Close Relationships–Short Form (ECR-SF) | 1 |
| Mental Readiness Form (MRF) | 1 |
| Positive and Negative Affect Scale (PANAS) | 1 |
| Nested data structure of consultation | 1 |
| Swedish Occupational Fatigue Inventory (SOFI) NASA-TLX instrument | 1 |
| Speech Content Analysis | 1 |
| Interview: Transcript Analysis | 1 |
| Profile of Mood States 2 | 1 |
| Facial Recognition Software: Affectiva | 1 |
| Interview: Analysis based on transformative learning framework | 1 |
| Self-Report: Qualitative analysis | 1 |
| Profile of Mood States (POMS) | 1 |
| Watson and Friend's Fear of Negative Evaluation Scale | 1 |
| Visual Analog Scale on Stress | 1 |
| Fear of Death Scale | 1 |
| Visual analog scale on psychological preparation (VASpp) | 1 |
| Social Avoidance and Distress Scale (SAD) | 1 |
| The Fear of Negative Evaluation Scale (FNE) | 1 |
| Test of Self Conscious Affect | 1 |
| Experimental Shame Scale (ESS) | 1 |
| Heart Rate | 1 |
| Blood Pressure | 1 |

# References

1. Arksey H, O’Malley L. Scoping studies: towards a methodological framework. *Int J Soc Res Methodol*. 2005;8(1):19-32. doi:10.1080/1364557032000119616

2. Creswell JW. *Research Design: Qualitative, Quantitative, and Mixed Methods Approaches*. 4th ed. SAGE Publications; 2014.

3. Turner TL, Balmer DF, Coverdale JH. Methodologies and study designs relevant to medical education research. *Int Rev Psychiatry*. 2013;25(3):301-310. doi:10.3109/09540261.2013.790310

4. Bilgic E, Gorgy A, Yang A, et al. Exploring the roles of artificial intelligence in surgical education: A scoping review. *Am J Surg*. Published online November 2021:S0002961021006826. doi:10.1016/j.amjsurg.2021.11.023
